# A Proposal of a Motion Measurement System to Support Visually Impaired People in Rehabilitation Using Low-Cost Inertial Sensors

**DOI:** 10.3390/e23070848

**Published:** 2021-07-01

**Authors:** Karla Miriam Reyes Leiva, Milagros Jaén-Vargas, Miguel Ángel Cuba, Sergio Sánchez Lara, José Javier Serrano Olmedo

**Affiliations:** 1 Escuela Superior Técnica de Ingenieros de Telecomunicación, Universidad Politécnica de Madrid, 28013 Madrid, Spain; milagros.jaen@ctb.upm.es (M.J.-V.); ma.cuba@alumnos.upm.es (M.Á.C.); sergio.sanchez.lara@alumnos.upm.es (S.S.L.); josejavier@ctb.upm.es (J.J.S.O.); 2 Engineering Faculty, Universidad Tecnológica Centroamericana UNITEC, San Pedro Sula 211001, Honduras; 3 Networking Center of Biomedical Research for Bioengineering Biomaterials and Nanomedicine, Instituto de Salud Carlos III, 28029 Madrid, Spain

**Keywords:** absolute orientation, inertial sensors, orientation and mobility, visually impaired rehabilitation

## Abstract

The rehabilitation of a visually impaired person (VIP) is a systematic process where the person is provided with tools that allow them to deal with the impairment to achieve personal autonomy and independence, such as training for the use of the long cane as a tool for orientation and mobility (O&M). This process must be trained personally by specialists, leading to a limitation of human, technological and structural resources in some regions, especially those with economical narrow circumstances. A system to obtain information about the motion of the long cane and the leg using low-cost inertial sensors was developed to provide an overview of quantitative parameters such as sweeping coverage and gait analysis, that are currently visually analyzed during rehabilitation. The system was tested with 10 blindfolded volunteers in laboratory conditions following constant contact, two points touch, and three points touch travel techniques. The results indicate that the quantification system is reliable for measuring grip rotation, safety zone, sweeping amplitude and hand position using orientation angles with an accuracy of around 97.62%. However, a new method or an improvement of hardware must be developed to improve gait parameters’ measurements, since the step length measurement presented a mean accuracy of 94.62%. The system requires further development to be used as an aid in the rehabilitation process of the VIP. Now, it is a simple and low-cost technological aid that has the potential to improve the current practice of O&M.

## 1. Introduction

People with visual impairments face many daily challenges that limit their quality of life. These challenges include basic life activities such as finding and keeping a job, mobility, and displacement, using public transport, among others. When a person is born with a visual disability or suffers from a traumatism or disease that leads to a visual impairment, they must be assisted trough a rehabilitation process. During this rehabilitation process, the person is provided with tools to help them deal with their visual impairments with greater independence and self-confidence. Tools as learning braille, learning how to use a long cane, sightless feeding, also to optimize the use of residual vision and teaching skills in order to improve visual functioning in daily life as well as other daily activities as O&M trained by specialists [1,2,3,4,5,6]. This process of rehabilitation is specialized according to the cognitive capacities of each user, the regular rehabilitation programs worldwide, as reported by the World Blind Union, which includes several stages, such as activities of daily living services, career exploration services, travel-training services/O&M, and others [7]. In several references [6,8,9,10,11,12] the emphasis and importance of the O&M service and training in order to improve the quality of life, is widely emphasized [5,13]. Therefore, there is a specific health discipline in charge of the study, development, and improvement of the O&M training in VIP [14,15,16]. The latest report of the international approaches to rehabilitation programs from the World Blind Union [7] presents two important challenges on which this project was motivated: (1) the limitation of resources to provide basic rehabilitation services and (2) transportation and geographic limitations, where many VIP must displace themselves to other cities in order to access the rehabilitation services which, in some cases, is impossible for some VIP.

A fundamental part of the mobility training is the use of the long cane, the VIP should learn how to hold it correctly, how to grip it, how to walk with it and sweep it in order to detect obstacles, different techniques of exploration, and other parameters according to the complexity of the environment in which the VIP will navigate [17,18]. This training is usually done in person with an O&M specialist, which, as mentioned before, leads to an accessibility problem in rural communities, also it compromises the rehabilitation duration, as well as the number of VIP that can be rehabilitated at the same time. In this training, depending on the scenario there is a recommended technique and according to the complexity and advances of the training, the scenarios will change [19]. However, the parameters for evaluation of the correct use, regardless of the change of scenario, will remain the same; this allows the possibility to register parameters and quantitative values of the motion of the person and the long cane [20], in order to support the O&M training in the rehabilitation processes.

According to the literature, a diversity of technological proposals have been designed for orientation and mobility, such as ETAS (Electronic Travel Aid Systems) [2], focused on obtaining information from the environment and providing it to the visually impaired in order to assist them in autonomous navigation. There have been many attempts to enhance the long cane with technology [21,22,23,24,25]. These systems are developed from technologies such as Global Positioning System, BLE beacons, RFID or radio frequency identification, to obtain information on position and displacement and optical sensors (RGB-D cameras, laser), inertial sensors, speed sensors among others for obtaining information regarding object detection [26,27,28,29,30,31,32]. However, the use of any of these ETAS requires previous O&M training [33,34], leading to an existing gap, which is the development of assistive technologies specifically focused on evaluation and assistance of the training process, so it can be more accessible for users.

Three articles of assistive rehabilitation tools for O&M were found in the literature; Schloerb et al. [35] developed a virtual environment system named BlindAid, created in order to enhance the O&M training. This is a software with haptic and auditory feedback in which the user can virtually visit different unknown places in order to create cognitive mental maps of the representation of these places. Oliveira et al. [36] created a programming language named GoDonnie, to be used as a tool to aid in the resolution of spatial problems involving O&M. This programming language was developed considering the criteria of accessibility and usability for VIP, with the assumption that by using GoDonnie, the user could improve programming and O&M skills, since the users are able to create mental maps of the environments and related objects. On the other hand, Gong et al. [37] developed HeliCoach an O&M training system created to help VIP to train the ability of audio orientation. This training environment is composed of a drone, which moves through 3D space and is used as a sound source. It is composed of a belt with a set of vibration motors for haptic feedback, the belt also contains an BNO055 IMU and six vibration motors controlled by an Arduino DFRobot Leonardo + Xbee. In this system high accuracy indoor localization system is needed for the perspective-driven interaction. For this goal, Ultra-Wide Bandwidth Microwave is used: the system uses four base stations and two tracking tags which are embedded into the drone and the cap of the user, respectively.

In comparison to the mentioned developed technologies, the aim of this research was to develop a simple-architecture hardware system using low-cost inertial sensors for data acquisition and test its reliability in the quantitative analysis of the parameters evaluated in the rehabilitation process of VIP by obtaining metrics that the O&M specialists personally examined to aid the rehabilitators during current practice of O&M while training travel techniques.

The system can provide information about the hand grip rotation, the safety zone, the hand height during the travel techniques, amplitude and patterns of the sweeping, and gait parameters with a high accuracy using only two inertial sensors.

Technologies based on inertial measurement unit sensors (IMU) are used in a large and ever-growing number of applications, such as intelligence guidance, self-driving robots [38,39], full body motion tracking [40,41,42,43] and navigation [26,44,45,46]. An accelerometer measures the external specific force acting on the sensor, which consists of both the sensor’s acceleration and the acceleration due to the earth’s gravity. A gyroscope measures angular velocity: the rate of change of the sensor’s orientation. Thus, the integration of gyroscope measurements provides information about the orientation on the sensor. Magnetometers complement accelerometers by providing sensor heading (orientation around the gravity vector), which is information that accelerometers or gyroscopes cannot provide. With the fusion of accelerometer, gyroscope and magnetometers, the orientation is estimated based on the direction of the magnetic field [39,44]. In the system presented in this paper, the parameters of O&M are calculated using absolute orientation values of the sensor fusion provided by the BNO055 IMU module. Note that the present article is an extended version of [47], where the algorithms to measure amplitude of the sweeping techniques and the orientation of the long cane were tested with 97% and 98% accuracy, respectively.

## 2. Materials and Methods

A tool was developed to evaluate the rehabilitation parameters during the experimental procedure. For the data acquisition an Arduino MKR1010 microprocessor was used with two 9DOF BNO055 IMU Bosch sensors. One sensor placed on the outer side of the leg of each participant and the other on the higher part of a 117 cm long cane. Serial communication was done via I^2^C protocol at a sample rate of 0.01 s. In order to remove noise components from the signal, a low pass filtering was performed, with a cutoff frequency of 20 Hz. The microprocessor was wired to a SD card module via SPI protocol and to two push buttons settled as input parameters to control the acquisitions manually. With the use of the Euler roll angle $\theta_{leg}$ and the interpretation of step detection according to the values of the filtered absolute orientation, an algorithm was developed to calculate step length using the local coordinates of the sensor placed in the leg. Additionally, to obtain the sweeping metrics with the local coordinates of sensor placed in the cane, the Euler roll $\varphi_{cane}$, pitch $\theta_{cane}$ and yaw $\gamma_{cane}$ angles were used to provide the grip rotation, the safety zone metrics and sweeping characteristics consecutively.

For the experimental procedure, the acquisitions were performed with 10 blindfolded volunteers. First, the volunteers were instructed and trained for each travel technique while sighted. A floor carrel was marked for the sweep training with an amplitude of around 1 m, they were asked to train each technique walking 20 steps three times. After that, they were blindfolded and asked to perform the travel techniques when displacing around 20 steps in the indicated direction, as described in Table 1. Each acquisition was repeated blindfolded three times, obtaining nine comparative metrics for each participant. The total time and displacement were measured using a 50 m measuring tape and a chronometer. This value served as references values to evaluate the accuracy of the measured gait parameters.

## 3. Results

### 3.1. Measurement of the Hand Height and the Safety Zone

**Figure 1:**
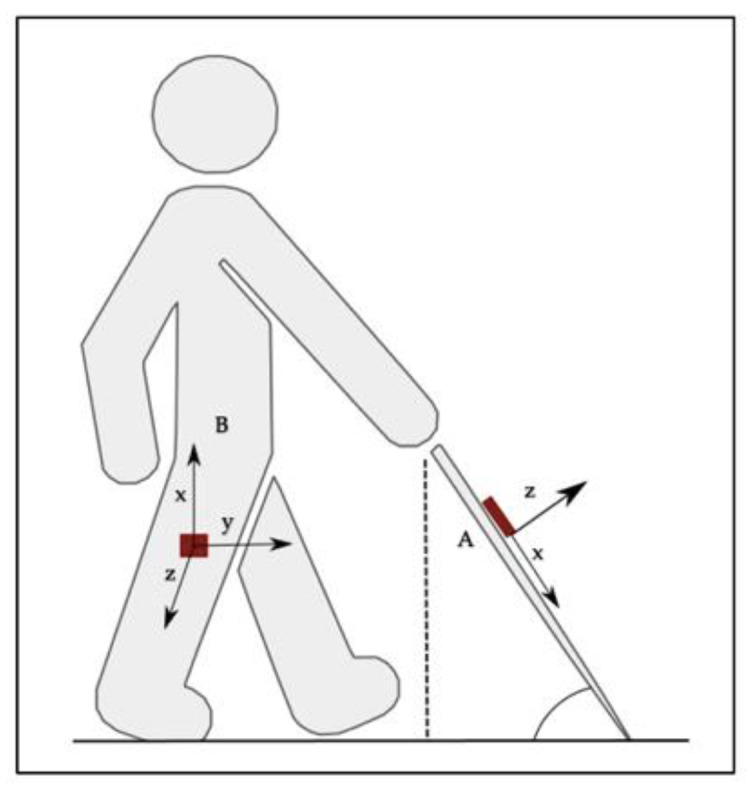
Local coordinate system of the sensor placed on the cane (A) and local coordinate system of the sensor placed in the leg (B).

The Hand Height (HH) and Safety Zone (SZ) are reference parameters to evaluate the reaction distance in O&M, which refers to the warning distance provided by the cane of an object in one’s path, the time that is provided by the cane to be warmed about an object or danger [48]. By implementing trigonometrical ratios and using the local coordinates of the sensor, the pitch angle $\theta_{cane}$ (which is the transversal axis, equivalent to the angle produced between the floor plane and the long cane) was continuously measured to obtain the height of the hand during and the distance between the tip of the cane and the leg, in the repetitions of the three different travel techniques. Being the HH, the opposite leg of the $\theta_{cane}$, the SZ then is the adjacent leg from the $\theta_{cane}$, as shown in Figure 1.

An extract of the measured values for HH and SZ for each subject is presented in Table 2. This value is compared with the real value (RV), which is the self-reported HH and the calculated SZ according to Pythagoras theorem. The mean value is the calculated media of the HH and SZ measurements within the nine travel technique acquisitions. The values of standard deviation (SD) and %Error vary for each subject. The major precision and accuracy obtained was with S01, being the standard deviation of only 1.39 cm, which represents 1.46% of the mean HH and 1.95 cm which represents 2.83% of the mean SZ and the %Error of 0.63% and 1.37%, respectively. Additionally, S09 presented a very low %Error, however a high SD (6.15) which together with S05 presented the less precision on repeatability, the SD being 5.03% of the mean HH and 8.60% of the SZ. On the other hand, the lower accuracy was shown by S06, followed by S07 and S08 with a %Error of 4.62% in the HH and 4.86% in the SZ measurement. Finally, a media accuracy of around 97.62% was obtained by joining all the subjects in both measurements proving that the algorithm applied is reliable to measure these O&M parameters using absolute orientation angles.

### 3.2. Measurement of the Grip Rotation

A proper grip was one of the first parameters to be observed by the rehabilitators during the very first stage of the O&M training. With the inertial sensors, is not possible to analyze all the characteristics of the grip, but it is possible to determine the variation of the rotation of the cane which is the consequence of the grip rotation by analyzing the absolute orientation angles, as shown in Table 3. In this table, the SD in degrees for each travel technique by subject was calculated and presented. For this, it was taken into account the total raw data of the roll angle $\varphi_{cane}$, which according to the local coordinates of the placed sensor represents the rotation of the grip of the user during the development of the travelling techniques. As shown in the Table 3, this value can be representative for technical analysis of the performance of the traveling techniques independently of the stage of and scene in which the user is being rehabilitated. It can also provide a numerical representation to establish what is considered as adequate and acceptable grip rotation according to each travel technique.

Note that the variation of the values represents the percentage of rotation of the grip during each experiment which means that each column represents how much variation in the rotation of the hand occurred during the experimental acquisition. In the results, it can be observed that S04 and S10 present less grip rotation in the 2P and 3P techniques, which is an indication of a better execution than for instance for S03 and S09. This is direct indication for the specialist to determine which is the acceptable percentage of rotation for each travelling technique and which technique is more appropriate for the visually impaired; it can also allow to have a tracking of the performance during the rehabilitation stages.

### 3.3. Representation of the Sweeping

**Figure 2:**
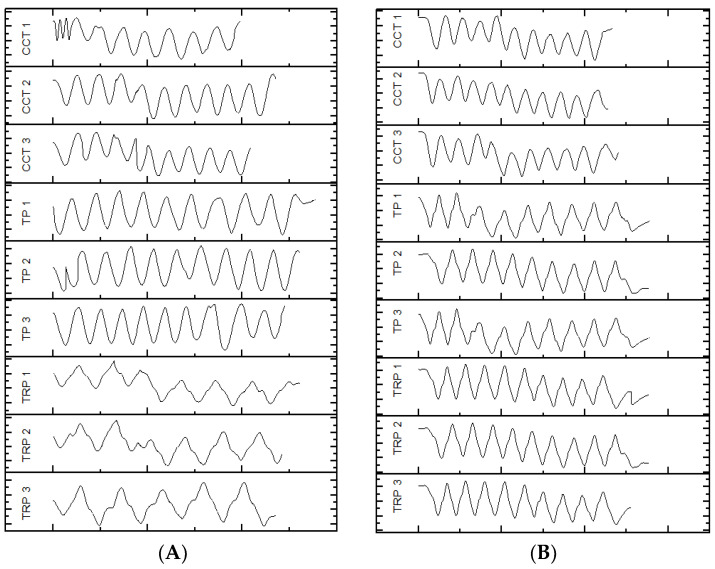
Sweeping preview ($\gamma_{cane}$) in the 20-step displacement for each travelling technique, S05 (**A**) and S06 (**B**).

In [47], it was clearly demonstrated that using absolute orientation angles was reliable to measure the amplitude of the sweepings with the long cane. As described by Blasch and LaGrow [48], the performance of the O&M rehabilitation can be evaluated in terms of “coverage” provided by the long cane, where a full coverage includes, for instance, object preview: the capacity to identify objects in the path of travel with a correct sweeping of the long cane. As the carried out traveling techniques consists of sweeping oscillatory movements, by extracting the motion of the yaw angle $\gamma_{cane}$, it is possible to graphically represent the movement of the long cane beside the value of the sweeping amplitude, as shown in Figure 2.

This graphical representation is indispensable in order to have an estimation of the performance of the travelling techniques while the user is in training, since it is a detailed characterization of the movement of the cane in each millisecond for the dynamic conditions. Additionally, it can help the rehabilitators to evaluate the coverage that is being provided in that moment of the execution of the travelling techniques. As well, for the user to self-correct any lack of coverage with immediate feedback to prevent an accident while correcting the amplitude and execution of the sweeping during the training. It can also help the user and the rehabilitator to quantitatively determinate which is the most appropriate travelling technique for the user. As shown in Figure 2, many differences are observed in the development of the traveling techniques for two subjects (A and B) with the same characteristics. This brings us to one last advantage of this tool, which is the possibility to register the performance of each user during the entire rehabilitation process for future data analysis.

### 3.4. Measurement of Gait Parameters

In terms of coverage, an appropriate gait is crucial for the development of O&M abilities [49], therefore during the O&M training, the gait velocity and the stride length is being constantly visually evaluated by the rehabilitator. With this tool, the method to evaluate the step length for calculating the gait parameters (Stride Length and Gait Velocity) was developed using also absolute orientation angles. With the inertial sensor placed on the outer side of the leg, with the same local coordinates as the sensor placed in the long cane, the pitch angle was used to calculate the step length in a walking cycle and two of the travelling techniques (see Figure 1). The step length was calculated in an algorithm averaging the estimation of the displacement of the leg during the gait cycle following the difference of each peak-to-peak representation of the oscillatory movements of the pitch angle, where each peak represents the higher value of each phase in the gait cycle. Therefore, by knowledge of the leg length of each user, and constantly laying up the values of $\theta_{\mathrm{legmax}}$ and $\theta_{\mathrm{legmin}}$, the step length could be calculated using the following equation:

(1) $$\mathrm{SL}=2\times \sin\left(\frac{\theta_{\mathrm{legmax}}-{\;\theta }_{\mathrm{legmin}}}{2}\right)\times \mathrm{LL}$$

where SL is the length of the step and LL is the length of the leg of the user. The algorithm is capable of detecting if a step is being executed with the $\theta_{\mathrm{legmax}}$ and $\theta_{\mathrm{legmin}}$ thresholds. Table 4 summarizes the measurements obtained in each experiment. Note that the value of the measurement of the SL is an average of the three measurements obtained for each repetition and the mean difference (MD) in centimeters is measured with the resulting three values of the average.

The difference in centimeters between the actual value and the measured value is very low in most of the cases (2.704 cm–12.370 cm), which indicates that the system is also reliable to estimate the step length, however, in order to calculate traveled distances using this value, it is necessary to set the measurement error and thus dismiss the accumulated errors. This was not possible because there is an extended variation of the mean %Error of the measurement from one subject to other, from 1.07% to 15.06%. The reason for this variation is unknown, perhaps so the proposed method does not estimate hip displacement in the gait cycle. Another reason could be the reliance on the sensor decalibration, however, the accuracy of the absolute angles sensed varies very little with calibration but, as the step length values lie in the order of centimeters, this can be a factor affecting the variation of the %Error, which has a mean of 94.62%.

## 4. Discussion

Kim et al. [20], presented a quantification of the characteristics of long cane usage. In this work, similar parameters are evaluated in terms of the coverage of the travelling techniques in relation to the rotation angles of the movement of the long cane. However, to develop this study, optical tracking cameras were needed in addition to an inertial sensor placed in the long cane. The presented tool allows the dynamic quantification of the characteristics of the movement of the long cane with a lower cost dispositive and complexity and with high precision. With the inertial sensors and the presented metrics, it will be possible to obtain outcome measures as stride rate, gate velocity (meters per minute), and grip characteristics. Additionally, the provided coverage and long mechanics will allow interpretations of the sweeping characteristics as amplitude, frequency and the ability to detect obstacles in the path, as it has been done previously either with more complex acquisition systems [15,50,51], simulated [52] or in some cases manually [53].

The presented measure of the SL can be considered for the estimation of the gait parameters in O&M. Considering the limitations of the method, the most remarkable element of this tool is the fact that the system brings a measurement with the simplicity of one inertial sensor placed in the leg, using only one absolute orientation angle. Most of the algorithms found in the literature considered, beside orientation angles, acceleration values for step detection and calculation of displacement [54], as addition of at least another sensing method, which brings many other limitations and complexity in the development of the algorithm [34]. This article presents a simple method for computing clinically relevant gait parameters, with acceptable precision and accuracy, as in [55]. However, it is a fact that more precision can be obtained implementing a new method considering the details of the swing of the gait cycle or implementing artificial intelligence for instance [55,56].

Currently, a motion analysis device able to evaluate the percentage of coverage provided by a travelling technique according to specific parameters of a user cannot be found in the literature. The RoboCane software [48] was not successfully adopted by the O&M research community in the last decade. This software was designed to calculate the coverage according to direct measurement (manual) of the specific variables of the user. On the other hand, the proposed tool will allow the O&M specialists to have a real estimation of the coverage that the users are providing to themselves in dynamic conditions, which will also help them to be more objective in the evaluation of the O&M training. Among O &M specialists and researchers, it is known that there is no standardization in training methods, and these methods may vary according to the experience of each specialist. That is why research in O&M can also benefit from this tool. The development of the presented tool permits evaluating these mobility parameters independently of the environment complexity in which the training is gradually subjected [57], as it is a low cost portable device. Moreover, further development must be done to obtain more quantification characteristics of the O&M performance of the VIP. By adding one more sensor to the body, for instance, parameters of postural stability and balance analysis can be obtained.

## 5. Conclusions

This article proposed a system able to overview the quantitative parameters of O&M for VIP, which are currently visually analyzed by O&M specialist during rehabilitation, such as sweeping coverage and gait analysis. The proposed tool provides motion analysis of the long cane and the leg by using placed low-cost inertial measurement unit sensors (IMU). The system was tested in laboratory conditions by six blindfolded volunteers following three travel techniques trained by VIP during rehabilitation. The experimental results indicate that this system is reliable for measuring grip rotation, safety zone, sweeping amplitude and hand position using orientation angles with an accuracy of 97%. In terms of future work, a further development is required for the system to be implemented as a rehabilitation aid. Thereby, a more precise method for step length must be obtained, since the mean %Error varies between 1.07% and 15.06% among experiments. Also, more parameters of O&M can be analyzed using IMU’s absolute angles, including postural stability and balance analysis. Finally, as the main purpose, the proposed system is a new, simple and low-cost technological aid that that has the potential to improve the current practice of O&M.

## Figures and Tables

**Table 1:** Description and representation of the top view of the travel techniques for the experimental evaluation of the developed system.

Constant Contact Technique (CCT)	Two Points Touch Technique (2PT)	Three Points Touch Technique (3PT)
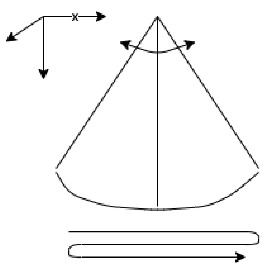	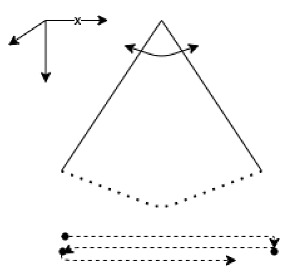	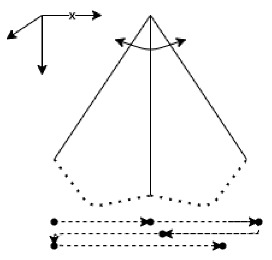
The CCT travel technique consisted of sweeping the long cane on the floor between two points with constant contact with an approximate amplitude of 1 m in order to provide coverage of the walking path.	The 2PT travel technique consisted of sweeping the long cane on the floor between two points taking the cane off the ground and creating an arc of around 5 cm, with an approximate amplitude of 1 m.	The 3PT travel technique consisted of sweeping the long cane on the floor between three points. One point on the left, one on the center and one on the right. Taking the cane off the ground in each point and creating an arc of around 5 cm.

**Table 2:** Extract of the measured Hand Height and Safety Zone and statistic characteristics.

	RV cm	Mean cm	SD cm	%Error	RV cm	Mean cm	SD cm	%Error
Hand Height (HH)					Safety Zone (SZ)			
S01	94.00	94.59	1.39	0.63	69.66	68.71	1.95	1.37
S02	89.00	90.17	3.17	1.31	76.00	74.34	3.77	2.18
S03	95.00	94.28	3.37	0.76	68.29	69.35	5.03	1.55
S04	86.00	82.64	2.60	3.90	79.32	82.68	2.59	4.24
S05	94.00	92.56	4.66	1.53	68.66	71.04	6.11	3.47
S06	86.00	82.03	3.75	4.62	79.32	83.17	3.54	4.86
S07	87.00	90.85	2.38	4.43	77.10	73.50	2.86	4.68
S08	88.00	84.13	2.51	4.40	78.23	80.21	4.35	2.54
S09	82.00	81.86	6.15	0.17	83.46	83.08	6.32	0.46
S10	83.00	81.15	2.50	2.23	82.46	84.19	2.39	2.09

**Table 3:** Standard deviation of the measured grip rotation for each subject in the acquisitions of the different travelling techniques.

SD in Degrees														
S01			S02			S03			S04			S05		
CCT	2PT	3PT	CCT	2PT	3PT	CCT	2PT	3PT	CCT	2PT	3PT	CCT	2PT	3PT
4.55	7.21	5.34	3.44	3.05	2.82	4.34	4.43	2.88	3.72	3.12	2.13	4.45	4.28	3.01
5.68	6.47	4.97	2.76	4.12	3.84	1.88	4.45	3.06	1.93	2.11	2.03	3.67	3.49	3.01
5.31	6.29	4.25	6.14	4.93	3.09	4.84	5.18	2.9	2.66	2.12	2.22	3.43	3.42	3.28
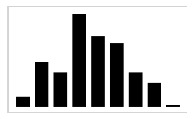			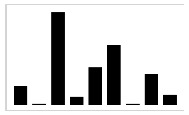			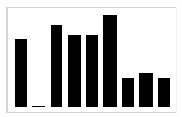			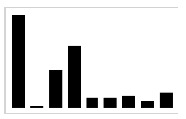			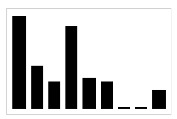		
S06			S07			S08			S09			S10		
CCT	2PT	3PT	CCT	2PT	3PT	CCT	2PT	3PT	CCT	2PT	3PT	CCT	2PT	3PT
3.1	3.92	3.92	8.37	5.61	3.92	5.68	3.63	3.5	7.43	7.43	6.19	2.17	2.88	2.47
3.47	3.06	3.06	7.4	6.55	4.72	6.8	4.16	2.91	8.84	7.12	4.4	6.15	2.98	2.41
2.97	2.84	2.84	7.97	6.24	4.5	6.18	5.1	3.11	6.13	7.15	5.75	4.35	3.1	2.21
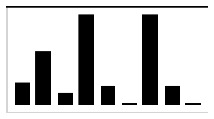			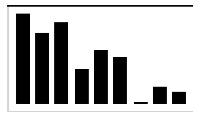			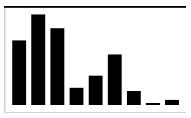			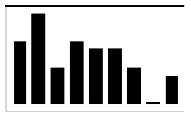			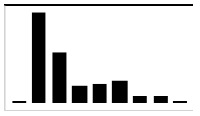		

**Table 4:** Step length measurement analysis.

Activity	SL m	RSL m	MD cm	SL m	RSL m	MD cm
	S01			S06		
W	0.553	0.500	7.046	0.560	0.546	2.769
CCT	0.517	0.405		0.490	0.443	
3PT	0.577	0.539		0.500	0.478	
	S02			S07		
W	0.553	0.551	2.704	0.678	0.625	4.224
CCT	0.590	0.601		0.731	0.716	
3PT	0.577	0.583		0.664	0.607	
	S03			S08		
W	0.447	0.432	4.937	0.567	0.502	12.370
CCT	0.530	0.534		0.581	0.536	
3PT	0.483	0.542		0.572	0.540	
		S04			S09	
W	0.603	0.592	3.333	0.520	0.502	3.047
CCT	0.563	0.603		0.500	0.536	
3P	0.580	0.550		0.536	0.540	
		S05			S10	
W	0.637	0.498	9.800	0.538	0.505	5.152
CCT	0.603	0.567		0.534	0.566	
3P	0.673	0.554		0.515	0.425	

W = walking.

## Data Availability

The data supporting reported results can be found in the following link: https://docs.google.com/spreadsheets/d/1-4NMnHSUrvO-NimwVJImX7G-dZibNwdqc_E2CO-AR00/edit?usp=sharing, accessed on 1 July 2021.
